# Maternity Care at the Intersections of Language, Ethnicity, and Immigration Status: A Qualitative Study

**DOI:** 10.1016/j.whi.2023.04.004

**Published:** 2023-05-25

**Authors:** May Sudhinaraset, Rebecca A. Kolodner, Michelle Kao Nakphong

**Affiliations:** a Jonathan and Karin Fielding School of Public Health, University of California, Los Angeles, Los Angeles, California; b School of Medicine and Health Sciences, George Washington University, Washington, District of Columbia

## Abstract

**Introduction:**

Women of color and immigrant women are more likely than US-born White women to report mistreatment and poor quality of care during their reproductive health care. Surprisingly little research exists on how language access may impact immigrant women’s experiences of maternity care, particularly by race and ethnicity.

**Methods:**

We conducted qualitative in-depth, one-on-one semi-structured interviews from August 2018 to August 2019 with 10 Mexican and eight Chinese/Taiwanese women (*n* = 18) living in Los Angeles or Orange County who gave birth within the past 2 years. Interviews were transcribed and translated, and data were initially coded based on the interview guide questions. We identified patterns and themes using thematic analysis methods.

**Results:**

Participants described how a lack of translators and language- and cultural-concordant health care providers and staff impeded their access to maternity care services; in particular, they described barriers to communication with receptionists, providers, and ultrasound technicians. Despite Mexican immigrants’ ability to access Spanish-language health care, both Mexican and Chinese immigrant women described how lack of understanding medical concepts and terminology resulted in poor quality of care, lack of informed consent for reproductive procedures, and subsequent psychological and emotional distress. Undocumented women were less likely to report using strategies that leveraged social resources to improve language access and quality care.

**Conclusions:**

Reproductive autonomy cannot be achieved without access to culturally and linguistically appropriate health care. Health care systems should ensure that comprehensive information is given to women, in a language and manner they can understand, with particular attention toward providing in-language services across multiple ethnicities. Multilingual staff and health care providers are critical in providing care that is responsive to immigrant women.

There are more than 44.7 million foreign-born individuals living in the United States, including 10.6 million living in California (Kids Count, 2020). More than 1 of 3 births (36%) in the state are to foreign-born mothers (Kids Count, 2020); however, immigrant women’s reproductive autonomy continues to be threatened. In recent years, fear of immigration enforcement policies have led to a mistrust and avoidance of maternal health services altogether (Rhodes et al., 2014) and anti-immigrant rhetoric and climate has corresponded with increases in preterm birth (Gemmill et al., 2019). Indeed, from 2007 to 2016, preterm birth increased by 2% among foreign-born women while declining by 11.5% among U.S.-born women during the same time period (Ratnasiri, Parry, Arief, DeLacy, Lakshminrusimha, et al., 2018), with similar trends for low birth weight (Ratnasiri, Parry, Arief, DeLacy, Halliday, et al., 2018). Moreover, studies also find that despite having lower rates of adverse birth outcomes, immigrant women, specifically those who are undocumented, are also more likely to experience higher rates of labor complications (Reed et al., 2005).

Women of color and immigrant women are also more likely to report mistreatment and poor quality of care during their sexual and reproductive health care. For example, Asian, Black, and Latinx women were twice as likely as White women to report that providers ignored them, refused to help when requested, or failed to respond in a timely manner (Vedam et al., 2019). Recent immigrants were also more likely to report instances of mistreatment compared with U.S.-born women (Vedam et al., 2019). Other data point to poor treatment during care for women with Medi-Cal (California’s Medicaid program), including having less consultation with providers, being less likely to be encouraged by staff to make their own decisions, and reporting more pressure to have a primary cesarean compared with women with private insurance (Declercq et al., 2020). Lack of patient-centered care among Hispanic women influenced their ability to understand information during prenatal visits, ask questions about their prenatal care, and desire to return for future appointments (Tandon et al., 2005). Few studies have focused on immigrant women in particular.

Reproductive autonomy is defined as the power to control one’s reproductive intentions, including contraceptive use, pregnancy timing, childbearing, or seeking an abortion (Upadhyay et al., 2014). Experiences in maternity care settings have the potential to impact future reproductive decision-making. Barriers to language access remain one of the most significant factors curtailing efforts to achieving reproductive autonomy among immigrant women (Ahmed et al., 2008; Ardal et al., 2011; Gagnon et al., 2010; Higginbottom et al., 2014; Ng & Newbold, 2011; Schapira et al., 2008). There is a need to move beyond understanding language needs as an individual-level challenge rooted in concepts of culture and acculturation (Viruell-Fuentes et al., 2012); that is, language should not be dismissed as an inherent cultural difference that cannot be overcome with structural change. The context of historical injustices and coercive policies targeting non–English-speaking immigrants in part relied on immigrants’ lack of language access and informed consent in reproductive health care (Stern, 2005).

Language access is defined as providing individuals with limited English proficiency (LEP) access to the same services provided to English speakers, in their language. California law mandates translated written materials and interpretation services for spoken interactions be provided at each point of service to those with LEP. For immigrants with LEP, challenges may include poor patient-provider interpersonal relationships, lack of fully informed consent for procedures or medication, and experiences of mistreatment and disrespect. Thus, in order to achieve person-centered reproductive health care for immigrant women, it is critical to consider the quality of maternity care at the intersection of language access, ethnicity, and immigration statuses. The objective of this study was to examine how language access may influence immigrant women’s experiences and quality of care. This study focuses on Mexican and Chinese immigrant women, two of the largest immigrant populations in the United States, to understand intersections across ethnicity and immigrant status.

## Methods

### Study Participants and Recruitment

The Research on Immigrant Health and State Policy Study is a convergent parallel mixed-methods study examining the lived experiences of Latino and Asian immigrants in California. Semi-structured, in-depth interviews were conducted to qualitatively examine the influence of state policies and immigration status on the experiences of accessing health care. This article examines a subset of qualitative interviews (*n* = 18) from the broader study that speci_fi_cally focus on women_’_ experiences of maternal health care.

We used a purposive sampling strategy based on ethnic and geographical criteria using input from the study’s Technical Advisory Committee. We decided to focus on a single group from each of California’s large and heterogeneous Asian and Latino immigrant populations to examine and understand key experiences for populations that share similar migration trajectories, immigrant statuses, and patterns of racialization. We also consulted with members of the study’s Community Advisory Board and other immigrant health researchers to select specific immigrant subgroups, bearing in mind population size, language, and social network considerations. We thus selected Mexican and Chinese (including those from Hong Kong and Taiwan) immigrant groups because they 1) represent two of California’s largest Latino and Asian subgroups, respectively (IPUMS, 2020); 2) similarly share a long history of migration to the United States and California, including traditionally migrating for economic reasons and exclusion by federal immigration law (Lee, 2002); and 3) have both experienced racialized exclusion (e.g., been perceived as a threat to dominant culture or labor competition), although patterns have been distinct. In addition, these groups include a diversity of immigration statuses, including those who were undocumented, with low rates of naturalization. Recognizing the influence of the local context, we sampled participants from two geographic locations that differed by local response to immigrants: Los Angeles and Orange Counties. Although both counties have large immigrant populations, Los Angeles is among California’s top counties in inclusive messaging, immigrant-serving organizations, and other “warmth of welcome” indicators, whereas Orange County is among the last (Pastor et al., 2012).

Eligibility for the study included identifying as 1) Chinese (including from Taiwan) or Mexican immigrant women; and 2) recently pregnant with a live birth within the past 2 years. Potential study participants were identified through study staff and referred through community partners, including community organizations, consulates, religious organizations, and snowball recruitment by participants.

Interviews were conducted from August 2018 to August 2019 by nine bilingual interviewers who were trained in qualitative interview methodology and techniques. Before study participation, respondents were informed about the purpose of the study and verbally provided informed consent. In-depth, one-on-one semi-structured interviews were conducted in person or over the phone in English, Spanish, or Mandarin depending on participant preference. Because interviews discussed private and personal information, confidentiality and privacy were ensured for all interview settings and data collection. Participants’ contact information was used for recruitment purposes only and destroyed on interview completion. Pseudonyms were used for all participants. Interview guides were structured in two parts: 1) the participants’ experiences of living in the United States as an immigrant (e.g., challenges, sources of support, and legal status changes), and 2) experiences navigating health care (e.g., health issues and experiences seeking care [or not]). In the second part, interview guides asked participants to describe their experiences and challenges during pregnancy, childbirth, and postpartum, including experiences with maternal health care. Probes were used to elicit details about interactions with the health care system, such as interactions with providers, perceptions of quality of care, and access to services. Interviews were audio recorded, transcribed, and stored anonymously. Participants were thanked for their participation with a $25 gift card.

### Data Analyses

Interview transcripts were uploaded to Dedoose Version 8.0.035 (SocioCultural Research Consultants, LLC, Los Angeles, CA), a qualitative data analysis software program, for coding. Mandarin interviews were translated directly into English and coded with interpreter notes to maintain precision and accuracy of translation while explaining cultural expressions and idioms. Spanish transcripts were coded in Spanish and specific excerpts translated into English for further analysis. Coding was performed by 11 members of the research team, 9 of whom were also interviewers. The codebook was developed by using an initial coding schema based on interview guide questions, then was iteratively expanded by constantly comparing themes and categories (Corbin & Strauss, 2008; Merriam & Tisdell, 2015). Interrater reliability tests were performed in Dedoose on a subset of most frequently used codes. Rater agreement across codes was measured by pooled Cohen’s Kappa statistics, which ranged from 0.80 to 0.96, indicating very good agreement (Landis & Koch, 1977). Throughout the coding process, the research team met weekly to discuss and reconcile differences, documenting all changes. Matrices were developed using the characteristics of ethnicity, county, and immigration status and themes such as quality of care, wait times in health care, interaction with prenatal doctor, and unfair treatment. We used thematic analysis to identify themes and patterns in transcripts. Language emerged as a core category on which our themes were centered.

All study materials and procedures were approved by the Institutional Review Board (IRB) at the University of California, Los Angeles (IRB# 17–001352). Verbal informed consent was obtained from all participants involved in the study.

### Research Team and Reflexivity

The research team comprised 7 investigators and 11 research staff. The research team was a diverse group of researchers, promotoras, and students. Three members had personal experiences of immigration, and 4 members had personal experiences with childbirth. Given the diversity of experiences and perspectives within the team, we practiced reflexivity in our research and considered potential subjective influences or biases in 2 ways. First, following all interviews, interviewers wrote memos to document their thoughts and evaluate their own reactions. Memos were also written after transcription of interviews and periodically throughout the phases of translation and coding. Second, positionality and personal reflection of team members’ awareness of personal biases, attitudes, and social position were regularly discussed in weekly meetings.

## Results

The study sample included 18 women who identified as immigrants from Taiwan or China (*n* = 8) or Mexico (*n* = 10) and had given birth within the prior 2 years (Table 1)_. The average_ age was 36.4, ranging from 26 to 46. Average time since arrival in the United States was 16.4 years (range 6–25 years). Participants were recruited from Orange County (*n* = 8) or Los Angeles (*n* = 10). The study sample included participants with different immigration statuses, including U.S. citizenship and legal permanent residence (the most protected statuses), visas or work permits, and undocumented status (the least protected).

Themes, categories, and subcategories are summarized in Table 2. Themes are organized in the table by relative saturation from qualitative interviews, presented in descending order.

### The Intersection of Language Access, Ethnicity, and Culture

Overall, almost all Mexican women described having access to Spanish-speaking providers and health care staff. Most Mexican participants reported seeing Spanish-speaking providers and attending Spanish-speaking clinics. In addition, some Mexican women explicitly mentioned their providers’ Latin American ethnicity when describing their satisfaction with care:

*The doctor received me with a smile, he is Cuban, a very good doctor. In fact, I moved all my children there…So with the help of the doctor and nurse I was able to relax, get out of the depression that was consuming me* (Mexican, Undocumented).

However, a subset of Mexican participants shared the sentiment that language was a main challenge in navigating health care and life in the United States. One Mexican woman shared the perception that some physicians require patients to speak English and that this was a violation of patients’ rights:

*There are, for example, doctors…who say, “oh you have to speak English,” or sometimes they don’t have things accessible to people who do not speak English…there are people who feel that they have the right, the right to say to people that they should speak English* (Mexican, Visa Holder).

The lack of translators and language-concordant health care providers and staff resulted in barriers to accessing maternity care services, such as communicating with receptionists, providers, and ultrasound technicians. For example, one Mexican woman said: “*English is one of the things that has closed doors to me in many places and made it quite difficult for me, appointments and all, because I do not speak English*” (Mexican, Undocumented).

Similarly, most Chinese women described how translation services were insufficient or inaccessible, despite hospital policies suggesting that these services should be provided.

*Then I asked him if there was any translation if the [expecting] parents needed it. They said no, so there was none there. It seems that they gave me a piece of paper on the hospital registration that said yes. However, if I asked them in person, they said no. I don’t know if there is any translation service or not, it seems that it is not very easily available* (Chinese, Permanent Resident).

Many Chinese women described a need for Chinese-language services.

Chinese participants also commonly mentioned language as an important factor when selecting providers. One woman described how her preference for Chinese-speaking providers developed because of her lack of understanding complex medical terminology:

*I didn’t plan to only look for Chinese doctors but I can’t understand English well…If you ask them more questions, they don’t—I mean they used too many terminologies, I can’t understand…And then later, I also changed to another obstetrician, who speaks my language. There’s a lot of terminologies* (Chinese, Permanent Resident).

Another woman shared a similar sentiment about the lack of language concordance in the medical setting when asked about any difficulty in the process of seeing a doctor (see Table 2, Culture, second quote).

Chinese participants also described a preference for culturally concordant providers to facilitate communication and a shared understanding. One participant who works as a community organizer described the decision-making process of selecting a provider (see Table 2, Language of provider, second quote). Across the sample, women’s expectations of care were influenced by their past experiences or perceptions. Across ethnicity, county, and immigration status, women shared the expectation that more difficulties arose for those who did not speak English. One Chinese participant acknowledged the importance of being able to communicate well and anticipated that new immigrants with low levels of English or acculturation would experience difficulties obtaining quality care, stating:

*If you don’t understand English, it’s kind of difficult to communicate. I would say, if you have been living in the U.S. for a longer time, or you’re able to communicate in English, there shouldn’t be a big issue for you. However, if you’re a new immigrant, or you’re not familiar with the lives in the U.S., you may feel a bit of difficulty then* (Chinese, Permanent Resident).

One Mexican woman who previously had a negative encounter with a physician caring for her newborn viewed a nurse’s supportive care and resource referral as a blessing from God. After experiencing negative encounters with the health care system, expectations of care held by this undocumented individual were so low that when she finally received quality care, it was viewed as a divine intervention as opposed to routine or standard.

*Thanks to God…We went to the hospital and there I saw the nurse who told me “What happened? Why are you like this?” I was very desperate, and I told her what the doctor had said and she told me, “No, don’t worry. You won’t go back to see that doctor.”…I am grateful to God that he had put her in my path because she was my angel* (Mexican, Undocumented).

### Language Access Is Associated With Experiences of Maternity Care

Difficulties in medical terminology oftentimes resulted in women receiving insufficient information that led to negative experiences with care and, in some cases, exacerbated health problems. One woman recalled how she negatively interpreted a provider’s assessment of her newborn because of language difficulties:

*That doctor told me, “You know what? Your baby is losing weight, he is not eating well, he is going to die.” That’s how he told me…I go out crying and she [mother-in-law] says to me, “What happened?” And I told her, “The doctor says that my baby is going to die…He says that my milk is useless and that I am going to kill him.” So then I began to enter into a very ugly depression* (Mexican, Undocumented).

This same participant also described how a lack of information resulted in a painful experience of care and feeling pressured into signing a document that violated her patient rights (see Table 2, Poor perceived quality of care).

In contrast, several women linked having language access with positive experiences with care. One woman reported:

*I tell my friends or people I know sometimes “Go to that clinic, they treat you good.” The same in the hospital, the hospital has a translator if you don’t speak English or if you don’t feel comfortable, you call and ask for a translator and they assign one. It’s really good service* (Mexican, Undocumented).

However, some participants described how seeking language-concordant providers led to negative experiences of care. Some Chinese women described that Chinese-speaking providers were in high demand, and their high patient volumes contributed to poorer care (see Table 2, Dissatisfaction with care). Although immigrant women sought out language-concordant providers to better navigate and understand processes of care, there were sometimes trade-offs with poorer treatment.

### Failure of Informed Consent and Reproductive Decision-making

Several Mexican women described negative experiences of being inadequately informed or consented for care. Some Mexican women identified failures of informed consent, including being partially informed, incomprehensible language, and inadequate description of available alternatives. One Mexican woman highlighted the illegible text size and lack of plain language in consent forms as a barrier to giving informed consent: *“We have to fill out forms that we don’t understand. Sometimes we have to sign consent forms with letters so small that it is hard to read even when I put on my reading glasses”* (Mexican, Citizen). She felt expected to sign consent forms that were full of technical language and *“concepts that we truly don’t understand.”*

Three Mexican women described instances in which they felt providers forced them to limit their fertility. One Mexican woman expressed feeling repeatedly excluded in clinical decision-making, resulting in an unwanted hysterectomy: *“They cut my womb, I couldn’t do anything…they [providers] told me, ‘I have to operate on you, no more kids. Your body says no more. I am going to give you the papers and you sign’”* (Mexican, Undocumented). She described how providers failed to provide comprehensive information about treatment options and disregarded her preferences about treatment. After birth, she was informed her body could not handle a future pregnancy due to uterine fibroids and, as a result, was recommended a partial hysterectomy. Although she consented to the sterilization as treatment, she reported substantial emotional distress because she was unaware that the hysterectomy would make her ineligible for the Family Planning, Access, Care, and Treatment (Family PACT) program that had provided her with reproductive care services (e.g., Pap smears, breast cancer screening services) and connected her with dental care and WIC benefits. She went on to say: *“I had therapy because I stayed very sad thinking that I wouldn’t have kids.”* Her narrative exposes providers’ failures to provide adequate information, appropriately educate the patient, and offer alternative forms of care, ultimately causing her regret and emotional distress.

Another undocumented Mexican woman discussed how doctors seemed judgmental about the number of children she had, resulting in the patient switching to a different provider (see Table 2, Pressure from provider, first quote). Shared in the experiences of these two women without legal immigration status are providers failing to offer comprehensive information and instead offering an unsolicited opinion in a way that belittled the reproductive decisions of these women.

### Strategies Used to Improve Language Access in Maternity Care Settings

Chinese and Mexican immigrant women used several strategies to overcome language barriers. Some women relied on informal support systems. For example, when translation services were lacking, two Chinese women brought their English-speaking husbands to translate. One woman relied on her partner until she could switch to a Mandarin-speaking provider. Similarly, one undocumented Mexican woman routinely brought her eldest daughter to interpret after encountering language barriers that left her feeling anxious. Despite being offered an interpreter, she preferred her daughter’s translation because she felt that the interpreter did not translate exactly what she was saying or ask all her questions.

Chinese participants also described additional strategies to overcome language barriers. When translation services were not offered in hospitals, Chinese women used cell phones to translate questions and unfamiliar terms in real time, often asking doctors and nurses to write down keywords (see Table 2, Use of technology).

However, this was not always sufficient, and some Chinese women described subsequently actively searching for Chinese-speaking providers. Compared with Chinese participants, Mexican women reported less use of technology for communication and, in one instance, reported lacking access to these resources, such as not owning a cell phone.

## Discussion

This study highlights the importance of addressing quality of maternity services at the intersection of language access, ethnicity, and immigration status. This study moves beyond understanding language proficiency, which has mostly been conceptualized as an individual-level factor rooted in concepts of acculturation, to one that focuses on language access as a structural-level resource that must be made available at each point in service to all immigrants who need it (Viruell-Fuentes et al., 2012).

Both Mexican and Chinese women described challenges in language and communication during their health care visits. Chinese women in this study described lack of translation services in hospitals. Even when hospital guidelines indicate they should be available, women described being denied services. Other studies among Chinese immigrants have similarly found an overwhelming preference for Chinese-speaking physicians, regardless of socioeconomic status (Kaiser Permanente, 2020). Mexican women in this study described many instances of failure of informed consent, including incomplete information or information given in a way that is not comprehendible or legible by participants. Mexican women in this study described instances in which they felt either forced or judged by providers to limit their fertility. Although only a few women discussed this, it is important to highlight given the history of racist policies against Latina immigrants in California (Stern, 2005). This includes the involuntary and widespread sterilization abuse in the early 1970s in Los Angeles among Latina immigrant women (Stern, 2005) and recent reports of forced hysterectomies in immigrant detention centers (Treisman, 2020). In our study, participants who were not provided the full information and range of alternative options described subsequent emotional and psychological distress. Combined, these findings highlight the importance of improving women’s quality of care from multiple domains, as access to translation services does not equate to women receiving full and comprehensive information. Women’s experiences described in this study underscore how providing immigrant women with clear, comprehensive information about their health, their care, and their rights as patients is crucial for ensuring women have agency over their health care.

It is also important to note individual and interpersonal resources that women deployed. Although Mexican women described adequate translation services in hospitals, Chinese women were more likely to describe using social resources in the form of informal networks, note-taking during visits, and using mobile phones. Other studies have similarly described the important role of husbands as health brokers (Sudhinaraset et al., 2016), and that paternal support during pregnancy decreased preterm birth (Ghosh et al., 2010).

### Limitations

This study has some limitations. It is important to note the differences across the Mexican and Chinese samples surrounding educational attainment status, documentation status, reason for migration, years in the United States, age, and language proficiency. The Chinese sample had a larger portion of women who migrated to the United States for educational purposes and had a lower average age. Mexican participants constitute all of the undocumented participants in the study, whereas the Chinese sample contains all of the legal permanent residents. In addition, only 37% of Chinese respondents, compared with 100% of Mexican respondents, had undocumented friends/family. It is plausible that the Mexican sample may have represented a more disadvantaged group in terms of documentation status and educational attainment, and therefore Mexican participants might have lower expectations of care and be less likely to report preferences.

Also, despite our efforts to select populations that represented two large yet distinct ethnic groups, a broad range of immigration statuses, and different local contexts, generalizability to other immigrant groups and other policy contexts (e.g., counties, states) is also limited. For example, immigrants whose primary languages are not widely spoken may experience greater difficulties in communication and access to care and those residing in other contexts (i.e., rural areas, areas with low density of immigrants) may form different preferences and perceptions of care. In addition, we were unable to discern the influences of socioeconomic status on experiences of care, largely because of sociodemographic heterogeneity across ethnicities in our sample. Last, although we recruited participants across immigration statuses, there were still insufficient numbers across each category to reach saturation. Future studies should examine the role of immigration and legal status in accessing language services and quality of maternity care.

### Implications for Practice and/or Policy

Reproductive autonomy cannot be achieved without access to culturally and linguistically appropriate health care. Public health practitioners should consider the importance of informal systems that support birthing people to achieve their health care goals during childbirth, including inclusion of family members as health brokers and the power of technology. Despite the promise of these interpersonal strategies, health care systems need to buffer these individual resources with broader language access services. Examples of structural-level strategies recommended to improve language access include policies such as the following: 1) requiring that medical interpreters be board certified; 2) providing Medicaid reimbursement to hospitals and providers for interpretation services; 3) requiring culturally sensitive programs during medical school training; and 4) allocating funds for perinatal education, including patients’ rights and how to navigate health care systems with LEP (Rea et al., 2021). Other systematic approaches in specific hospital settings include ensuring the coverage of diverse Asian languages, multilingual signage, and development of health care interpreter certification programs to train a diverse workforce (Kaiser Permanente, 2020). Our findings suggest that there is a need for translation services to be available and paid for by hospitals and clinics and information to be given in a way that is comprehensive and understood by those with LEP. Health care providers and staff should assess immigrant birthing people’s preferences around their fertility and provide adequate postpartum contraceptive counseling that recognizes the history of trauma. Hospitals should give all patients comprehensive information in a language and manner they can understand. Multilingual staff and health care providers are critical in providing care that is responsive to immigrant women and promoting reproductive autonomy.

## Figures and Tables

**Table 1:** Participant Characteristics

Demographic characteristics	Mexican *n* = 10	Chinese/Taiwanese *n* = 8	Total *n* = 18
Age, y			
Mean	38	34.4	36.4
≤30	2	3	5
31–40	2	4	6
>40	6	1	7
Legal status			
Naturalized citizen	1	1	2
Permanent resident	0	5	5
Visa/Work permit (including DACA)	3	1	4
Overseas-born citizen	0	1	1
Undocumented	6	0	6
Time in United States, y			
Mean	20	11.9	16.4
County			
Los Angeles	5	5	10
Orange County	5	3	8
Undocumented family or friends	10 (100%)	3 (37.5%)	13 (72.2%)

**Table 2:** Themes, Categories, and Subcategories, According to Level of Data Saturation by Theme, Listed in Descending Order

Theme	Category	Subcategory
Intersection of language access, ethnicity, and culture	Language concordance	Language of provider *“I didn’t plan to only look for Chinese doctors, but I can’t understand English well… If you ask them more questions, they don’t—I mean they use too much terminology, I can’t understand… And then later, I also changed to another obstetrician, who speaks my language… There’s a lot of terminology”* (Chinese, Permanent Resident). *“I think it depends on your acculturation level and your language level…But for the people I serve in the organization, it seems that most of them prefer to go to [a] Chinese hospital because of language barrier[s] and culture. For example, Chinese may use Chinese medicine, and then there may be some home remedies at home. But it is related to Chinese culture, such as pressing acupuncture points…”* (Chinese, Naturalized Citizen). Culture *“…the doctor received me with a smile, he is Cuban, a very good doctor. In fact, I moved all my children there…So with the help of the doctor and nurse I was able to relax, get out of the depression that was consuming me”* (Mexican, Undocumented). *“Not really, except the language barrier. Since the doctor is White, she speaks English. I mean I don’t have any problems communicating with other people in daily conversations. However, when it comes to medical settings, I just don’t know that much”* (Chinese, Work Permit). Translation services *“Then I asked him if there was any translation if the [expecting] parents needed it. They said, ‘no’ so there was none there. It seems that they gave me a piece of paper on the hospital registration that said yes. However, if I asked them in person, they said no. I don’t know if there is any translation service or not, it seems that it is not very easily available”* (Chinese, Permanent Resident).
	Communication difficulties	Limited English proficiency *“English is one of the things that has closed doors to me in many places and made it quite difficult for me, appointments and all, because I do not speak English”* (Mexican, Undocumented). Discussion of discrimination *“There are, for example, doctors…who say, ‘oh you have to speak English,’ or sometimes they don’t have things accessible to people who do not speak English… there are people who feel that they have the right, the right to say to people that they should speak English”* (Mexican, Visa Holder).
Language access is associated with experiences of maternity care	Experiences of care	Interaction with providers *“I liked the services, the doctor. Whatever I asked them [in English], they answered. If I had to change positions, they helped me. For example, there are belts that they put on you and I had to move, they told me, ‘wherever is comfortable for you, we will help you.’ And they helped me with everything I needed and if I asked for anything, they responded with how it was”* (Mexican, Work Permit). *Poor perceived quality of care* *“I don’t know how they treated me but the truth is I had a very bad experience. ..The dentist did not give me the anesthesia well, almost, and took it out red hot and I, pregnant, could not be given any more anesthesia. So it was a horrible experience… they [obstetrician’s office] made me sign a paper that I can’t change doctors because if I change I have to pay $50…Now I know it’s not true but at the time I didn’t know”* (Mexican, Undocumented).
	Consequences of poor communication	Description of well-being *“That doctor told me, ‘You know what? Your baby is losing weight, he is not eating well, he is going to die.’ That’s how he told me…I go out crying and she [mother-in-law] says to me, ‘What happened?’ And I told her, The doctor says that my baby is going to die…He says that my milk is useless and that I am going to kill him’ So then I began to enter into a very ugly depression”* (Mexican, Undocumented). Dissatisfaction with care *“There was one time I saw a doctor who speaks Chinese, but s/he has too many patients, like she’s working on an assembly line…But there were so many patients in line. No matter what kind of diseases you have, they just use one flow of process to settle you down”* (Chinese, Visa Holder).
Strategies employed to improve language access in maternity care settings	Informal support	Translation by family/friends *“For example, there are a variety of things in the pregnancy process. I do not understand some things, such as the obstetrician, I don’t know how to…I have to check with him. Well, there are still some obstacles in language and medical language, so sometimes I have to go with my husband, because he can say English well, so I take him with me”* (Chinese, Permanent Resident). *“If you ask them more questions, they don’t—I mean they used too much terminology, I can’t understand. Therefore, when my kid’s father has days off, I’ll go together with him”* (Chinese, Permanent Resident).
	Self-translation	Use of technology *“I wrote down all the keywords on a paper, and used my phone to check it. Haha! I don’t understand anything she said. I asked her to write it down on a paper. The doctor knew that, she just put every keyword down on a paper, same as the nurses. Whenever I didn’t understand, I just asked them to write [it] down for me… I had my phone in my hand, I could just check it there”* (Chinese, Work Permit).
	Altering health care	Switching providers *“There was one time I saw a doctor who speaks Chinese, but they had too many patients, like they are working on an assembly line. I feel like, since I have the health insurance, I can just go anywhere to see a doctor. It’s the same price. Therefore, I eventually found a White doctor in Newport…Right, for sure it’s more convenient to have someone who speaks Chinese…I don’t really like it, so I switched to my current doctor”* (Chinese, Work Permit). Seeking language-concordant providers *“And then later, I also changed [to] another obstetrician, who speaks my language.”* (Chinese, Permanent Resident).
Failure of informed consent	Decision-making	Forms and documents *“We have to fill out forms that we don’t understand. Sometimes we have to sign consent forms with letters so small that it is hard to read even when I put on my reading glasses”* (Mexican, Citizen). Pressure from provider *“The doctor started saying ‘Have you thought about having an operation [referring to a hysterectomy/sterilization]? You have to be operated on because you are quite old to continue having children. You don’t have to think about it, you have to do it’….She was mad and asked why I had so many kids at the age I was and I didn’t like that treatment”* (Mexican, Undocumented). *“They cut my womb, I couldn’t do anything… they [providers] told me, ‘I have to operate on you, no more kids. Your body says no more. I am going to give you the papers and you sign’”* (Mexican, Undocumented).
	Regret for clinical decisions made	Emotional distress *“I had therapy because I stayed very sad thinking that I wouldn’t have kids”* (Mexican, Undocumented). Unexpected consequences *“Now my big problem is when I gave birth to her, they operated on me. They did not explain it well that when I was operated on, I would not have [social] services available. I don’t want to say that I want everything free, but they made me feel like I don’t matter now that they’ve operated on me and I can’t have any more children”* (Mexican, Undocumented).
